# Mountains are not like poles for symbiotic and saprotrophic soil fungi

**DOI:** 10.1111/nph.70084

**Published:** 2025-03-25

**Authors:** Peter G. Kennedy, Matthew E. Smith

**Affiliations:** ^1^ University of Minnesota 1479 Gortner Ave St Paul MN 55108 USA; ^2^ University of Florida 2550 Hull Road Gainesville FL 32611 USA

**Keywords:** biogeography, elevation, fungal richness, latitude, species distributions

## Abstract

This article is a Commentary on Barbi *et al*. (2025), **247**: 295–308.

Understanding how soil fungal communities respond to environmental gradients is crucial for predicting ecosystem functions in a changing climate (Baldrian *et al*., 2023). In a study published in this issue of *New Phytologist*, Barbi *et al*. (2025; pp. 295–308) investigate how different fungal guilds – ectomycorrhizal (ECM), saprotrophic (SAP), and root endophytic (REND) fungi – are distributed across latitudinal and elevational gradients at 17 sites throughout Europe. This work provides a much‐needed perspective on whether mountains function as ‘latitudinal analogs’ for soil fungal communities and how different fungal lifestyles mediate biogeographical patterns. Their findings both confirm and challenge our prevailing understanding, particularly regarding the universality of the mid‐domain effect as an explanation for fungal elevational distributions.

*…the findings highlight the need to move beyond simplistic models that assume uniform responses across taxonomic or functional groups*.

This study employs a standardized, continent‐wide sampling protocol across sites spanning from Spain and Greece, in the south, to Iceland and Norway, in the north, ensuring robust cross‐site comparability. By applying various statistical models, including joint species distribution models, the researchers capture both guild‐level diversity patterns and species‐specific responses to both elevational and latitudinal gradients, with a specific effort toward disentangling the impacts of climate relative to other ecological variables. This rigorous approach, combined with thorough bioinformatic analyses, meets all the important benchmarks for assessing fungal community structure at large spatial scales.

All three fungal guilds displayed a significant trend in operational taxonomic unit (OTU) richness, but none were significant for both elevation and latitude (Fig. 1). This suggests that the responses to these two gradients are not direct analogs, despite sharing similar climatic and vegetational trends. SAP fungal richness significantly decreased with increasing elevation, while REND fungal richness significantly increased with increasing latitude. By contrast, ECM fungal richness displayed a nonlinear response, showing a significant positive unimodal relationship with elevation. Importantly, for all three guilds, there was no significant interaction between the effects of elevation and latitude on OTU richness. This finding contrasts with the idea that ‘mountain passes are higher in the tropics’ (Janzen, 1967); that is, high elevations at low latitudes present more significant physiological barriers to organisms than similar elevations at higher latitudes. This result does not, however, mean that climate was not an important predictor of fungal OTU richness, as multiple climate‐related variables were identified as significant explanatory variables of OTU richness in random forest models. Instead, this finding indicates that additional ecological variables beyond climate also strongly impact fungal guild spatial distributions.

**Fig. 1:**
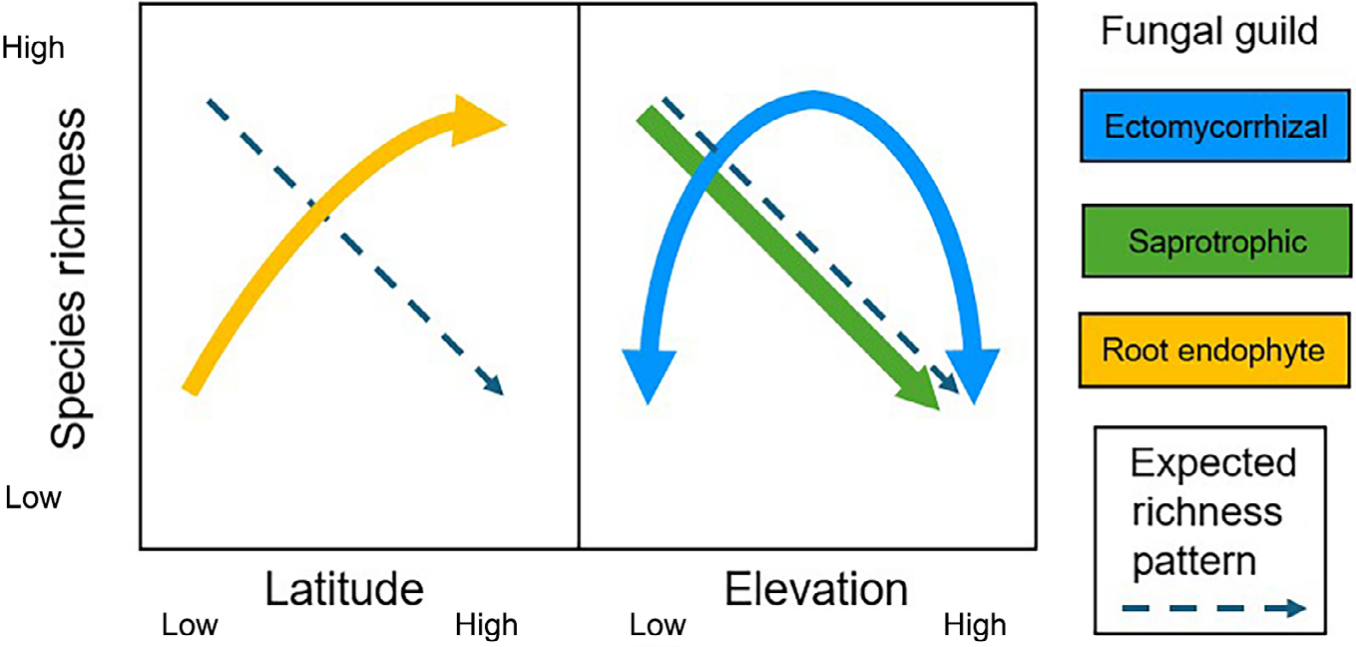
Summary of species richness trends by soil fungal guild across latitude and elevation relative to trends typically observed for plants and animals.

The range of richness patterns across guilds provides strong evidence that fungal species distributions are not the product of ‘null’ ecological processes. One well‐cited explanation for a unimodal richness pattern across a bounded domain is the mid‐domain effect (Colwell *et al*., 2000), which posits that because of hard constraints at each end of the domain (i.e. a species cannot have any of its range outside the domain), more species' ranges will overlap toward the center of the domain and thereby increase richness. While this study did find that ECM fungal richness was unimodal with elevation, the authors convincingly argue that the absence of similar patterns in either of the other two guilds indicates that fungal species distributions based on simple geometric space‐filling are not the norm. Interestingly, for ECM fungi, soil pH emerged as a significant elevation‐independent driver of species richness. It is well known that some evolutionary lineages of ECM fungi (e.g. Pezizales) respond favorably to higher soil pH (peak ECM fungal richness in this study was observed at *c*. pH 6), which hints at the importance of considering how the diverse evolutionary histories of different ECM fungal lineages might differentially impact their distributions (Petersen, 1985). The extent to which modifying soil pH might be used as a management tool to optimize or maintain ECM fungal richness is also an interesting possibility uncovered by these findings.

The current working explanation for why ECM fungal richness has a unimodal latitudinal pattern globally – that temperate forests have a unique combination of higher host phylogenetic richness and stem density than tropical and boreal forests (Kennedy *et al*., 2012; Tedersoo *et al*., 2014) – was partially controlled for in the study design. Here, ECM host tree species richness was held effectively constant at each elevation and latitude, with no more than two species of ECM trees per site. However, whether the phylogenetic richness of the hosts, which increases ECM fungal richness (Nguyen *et al*., 2016), or ECM host stem density including understory shrubs (Tedersoo *et al*., 2024), was higher at mid‐elevations was not presented. Support of nonhost‐related factors driving ECM fungal richness elevation patterns has been previously demonstrated by Truong *et al*. (2019), who examined ECM fungal communities across a range of elevations but with a single host tree species (*Nothofagus pumilio*) in Patagonia. Although they found a similar unimodal richness pattern for ECM fungi as in this study, they also showed that fungal‐associated soil enzyme activity did not change with elevation. That difference was attributed to a shift in ECM fungal composition toward specific taxa known to produce proteolytic enzymes at higher elevations. This study by Barbi *et al*. does not address whether there were similar shifts in fungal community composition with elevational gradients in Europe, but the results of Truong *et al*. (2019) suggest that documenting changes in richness alone is not sufficient to assess soil fungal community impacts on C and N cycling across environmental gradients.

As with all scientific studies, the results of this study generate as many questions as they answer. For example, do increases in REND fungal OTU richness at higher latitudes correspond with any shifts in their facultatively saprotrophic lifestyle? For SAP fungi, given the wide diversity of substrates targeted, are there cryptic richness patterns within litter vs wood vs soil saprotrophs by elevation or latitude? For ECM fungi, are there higher‐level taxonomic changes across these two gradients? For example, were there notable changes in richness for particular evolutionary lineages of ECM fungi across latitudinal or elevational gradients that could help to inform the larger pattern? ECM fungi share a dependence on their host plants for carbon, but they are morphologically and physiologically diverse, have evolved > 65 times across a wide geological timescale (Tedersoo & Smith, 2013), and have diverse geographical origins, including both temperate and tropical zones (Looney *et al*., 2016). As such, it will be important in future studies to more fully integrate how this evolutionary diversity also shapes the distribution patterns and functions of distinct ECM fungal lineages.

Perhaps most intriguingly, is whether the authors' suggested explanation for the decreased ECM fungal richness at low elevations is correct. They speculate that the higher interval and longer history of human disturbance at low‐elevation sites may erode ECM fungal OTU richness, similar to declines in ECM fungal richness across rural–urban gradients (Tatsumi *et al*., 2023). Testing this hypothesis will require additional study, perhaps by surveying forests with different intensities and intervals of logging history (Kebli *et al*., 2012). Regardless of these additional questions, this study provides an important step forward by demonstrating that different fungal guilds follow distinct patterns along both elevation and latitude gradients. The standardized sampling and processing approach strengthens confidence in the results, and the findings highlight the need to move beyond simplistic models that assume uniform responses across taxonomic or functional groups. As global change continues to reshape ecosystems, studies like this will be invaluable for predicting how soil fungal communities – and the ecosystem functions they support – respond to environmental shifts.

## Disclaimer

The New Phytologist Foundation remains neutral with regard to jurisdictional claims in maps and in any institutional affiliations.
